# Bacteriological methods as add on tests to fine-needle aspiration cytology in diagnosis of tuberculous lymphadenitis: can they reduce the diagnostic dilemma?

**DOI:** 10.1186/s12879-014-0720-z

**Published:** 2014-12-31

**Authors:** Ketema Abdissa, Mulualem Tadesse, Mesele Bezabih, Alemayehu Bekele, Ludwig Apers, Leen Rigouts, Gemeda Abebe

**Affiliations:** Department of Medical Laboratory Sciences and Pathology, Jimma University, Jimma, Ethiopia; Department of Clinical Sciences, Institute of Tropical Medicine, Antwerp, Belgium; Mycobacteriology Unit, Department of Microbiology, Institute of Tropical Medicine, Antwerp, Belgium

## Abstract

**Background:**

The diagnostic accuracy of fine-needle aspiration (FNA) cytology for the diagnosis of tuberculous lymphadenitis (TBLN) is confounded by mimicking cytomorphologic disorders. The objective of this study was to determine whether supplementing FNA cytology with bacteriological methods improves the overall accuracy of TBLN diagnosis.

**Methods:**

Two hundred presumptive TBLN cases were included in the study. FNA specimens were collected and examined for cytomorphologic changes, for acid-fast bacilli (AFB) by microscopy and for mycobacterial growth on culture. Culture was done using Lowenstein-Jensen (LJ) medium and mycobacteria growth indicator tube (BACTEC MGIT 960 TB detection system). Differentiation between *M. tuberculosis* complex (MTBc) and non-tuberculous mycobacteria (NTM) was done by using 500 μg/ml para-nitrobenzoic acid (PNB) susceptibility testing.

**Results:**

Cytomorphology detected TBLN among 80% (160/200) of the presumptive cases. Culture results were available for 188 cases. Twelve samples were excluded due to contamination on both culture methods. Culture confirmed cases accounted for 78% (147/188) of which MTBc constituted 97.3% (143/147). Among presumptive cases, classified by FNA cytology as ‘abscess’, 11 were culture positive. Microscopy detected 31.3% (46/147) of culture confirmed mycobacterial lymphadenitis of which 11% (4/37) were diagnosed non-suggestive for tuberculosis (TB) by FNA cytology. Compared to culture (LJ & BACTEC MGIT 960) and AFB microscopy as composite gold standard, FNA cytology had a sensitivity of 88.4% and a specificity of 48.8%. The positive predictive value was 86.1% while the negative predictive value was 54.1%. The confirming power and the ROC curve area was 1.73 and 0.69, respectively.

**Conclusion:**

FNA cytology showed a relatively high sensitivity but a low specificity. Combining bacteriological methods with FNA cytology in an endemic region like Ethiopia improves the overall accuracy of the diagnosis of mycobacterial lymphadenitis, which in turn may lead to better patient management.

## Background

According to the 2012 WHO report, the prevalence and incidence of TB in Ethiopia was 200/100,000 and 160/100,000, respectively [1]. Among the 22 high TB burden countries Ethiopia ranked seventh in terms of all forms of TB and third in terms of extra-pulmonary TB (EPTB). About 80% of EPTB in Ethiopia is TBLN [2]. In 2011, the Ministry of Health of Ethiopia reported 159,017 cases of TB. The proportions of smear-positive, smear-negative, and extra-pulmonary TB were 32.7%, 34.8%, and 32.5%, respectively [3]. A community based survey in Southwest Ethiopia indicated that the prevalence of lymph node TB was 58.0 per 100,000 population [4].

Even though the case detection rate of smear-positive pulmonary TB has slightly improved (from 31% in 1999 to 36% in 2010) [5], little attention was given to EPTB. Clinicians suspect that lymph node TB is over diagnosed, when the diagnosis is based on the national diagnostic algorithm. A study conducted in four different sites reported that only 78% of TBLN cases were culture positive [6]. Diagnosis by PCR revealed that 87.5% of TBLN cases identified by clinical and cytological criteria, were positive for mycobacterial DNA [7]. According to a study conducted in Northwest Ethiopia, successful treatment was achieved in only 24% of TBLN patients with a total death rate of 5.2% [8]. The study reported that the outcomes of other patients were unknown due to transfer to other health institutions in the region. Confirmation of the diagnosis merely relies on clinical evolution, which may lead to unnecessary treatment and further complications. Hence, it is important to supplement FNA cytology with better methods. We conducted a comparative study to determine whether supplementing FNA cytology with bacteriological methods improves the overall accuracy of the diagnosis of TBLN.

## Methods

### Study design and setting

A hospital based cross-sectional comparative study was done from April 1, 2012 to August 30, 2012 at Jimma University Specialized Hospital, Jimma zone, Southwest Ethiopia.

### Study participants and sample size

All patients visiting the pathology diagnostic unit for presumptive TBLN (lymph node enlargement) were considered as source population. Using the single proportion formula and taking 78% sensitivity for FNA cytology as screening tool [6], the required number of confirmed TBLN cases was determined to be 150. Taking a mean prevalence rate of 70% of TBLN among presumptive cases [6],[7],[9], 200 consecutive presumptive TBLN cases needed to be included in the study. Critically ill patients and patients already on anti-TB drugs were excluded from the study.

### Data collection

A structured questionnaire was used to collect socio-demographic and clinical data. Trained data collectors working at the pathology diagnostic unit collected the data. HIV test results were retrieved from the patients’ records after obtaining informed consents. The pathologist in charge collected FNA specimens. The first drop of the specimen was used for the cytomorphologic analysis and AFB microscopy. TBLN diagnosis by cytology was made by observation of the presence of epitheloid cell granulomas and caseous necrosis with or without Langhan’s giant cells [10],[11]. The remainder of the specimen was used for culture. Decontamination and digestion of the specimens was done with 0.5% N-acetyl-L-cysteine (NALC)-4% sodium hydroxide (Sigma Aldrich) -2.9% sodium citrate solution and subsequently concentrated by centrifugation at 3000 RPM for 15 minutes. Sediments were re-suspended in 1 ml phosphate buffered saline prior to inoculation on LJ slants (Becton, Dickinson and Company, Sparks, MD, USA) containing both pyruvate and glycerol. Additionally 500 μl was inoculated on BACTEC MGIT tubes (Becton, Dickinson and Company, Sparks, MD, USA) according to the manufacturer’s instructions. Differentiation of MTBc from NTM was done on LJ media containing PNB (500μg/ml). Strains growing on PNB containing media were considered as NTM. All procedures were done at Jimma University Laboratory of Mycobacteriology.

### Data analysis

Data were double entered using SPSS version 15.0 statistical software (SPSS Inc. Chicago, 2007) and exported to Stata 12 (Statacorp, USA). Descriptive analyses were done to depict the TBLN presumptive cases’ characteristics. Sensitivity and specificity of the different diagnostic methods (FNA cytology, AFB microscopy, culture on LJ, & BACTEC MGIT 960) were calculated using composite bacteriological methods as reference standard.

### Ethical consideration

Jimma University research ethics review board approved this study. Patients were included only after written consent was obtained. AFB microscopy and culture results were reported back to the clinicians and used for patient management.

## Results

Two hundred consecutive presumptive TBLN cases were included. Since 12 specimens were contaminated, results for 188 cultures were used for analysis. Female presumptive cases accounted for 55% (104/188) of the total study population. The mean age of the study participants was 29.3years ±14.4(SD) and 33.0% were in the age category of 16–25 years (Table 1). Among patients with known sero-status (n = 106), the HIV-TB co-infection rate was 8.9%. The majority of the cases presented with a lymph node swelling of less than one month. The cervical region was the most involved site (59% (118/200)) (Table 2).

**Table 1:** **Characteristics of study participants included in the study (n = 200)**

Patient characteristics		N (%)
**Age**	0-5	6 (3)
	6-15	22 (11)
	16-25	66 (33)
	26-35	54 (27)
	36-45	31 (15.5)
	46-55	13 (6.5)
	>55	8 (4)
**Sex**	Male	93 (46.5)
	Female	107 (53.5)
**FNA appearance**	Caseous	86 (43)
	Non-caseous	114 (57)
**Fever**	Yes	155 (77.5)
	No	45 (22.5)
**Night sweating**	Yes	139 (69.5)
	No	61 (30.5)
**Weight loss**	Yes	135 (67.5)
	No	65 (32.5)
**Loss of appetite**	yes	117 (58.5)
	No	83 (41.5)
**Cough of more than two weeks**	Yes	69 (34.5)
	No	131 (65.5)
**Lymph node mobility**	Yes	114 (58.2)
	No	82 (41.8)
**Presence of BCG scar**	Yes	87 (43.5)
	No	113 (56.5)
**Contact with chronic coughers**	Yes	47 (23.5)
	No	153 (76.5)
**Lymph node scar**	Yes	74 (37)
	No	126 (63)

**Table 2:** **Site of lymph node enlargement and FNA cytology result among TBLN suspects (n = 200)**

Dummy		FNA cytology result					Total
		Tuberculosis	Non - specific Abscess	Reactive LN*	Pyogenic infection	Other diagnosis	
**Lymph node site**	Cervical	95	6	6	7	4	118
	Axillary	27	4	2	3	2	38
	Inguinal	14	0	1	0	0	15
	Cervical and Axillary	11	0	0	0	1	12
	Cervical and Inguinal	1	0	0	0	0	1
	Axillary and Inguinal	1	0	1	0	0	2
	submandibular	11	3	0	0	0	14
**Total**		160	13	10	10	7	200

* = Reactive lymphadenitis; FNA = fine needle aspirate; TBLN = tuberculous lymphadenitis.

The prevalence of bacteriologically confirmed (MGIT 960 and/or LJ positive) mycobacterial lymphadenitis was 78% (147/188). MTBc was identified in 97.3% (143/147) of the culture-positive cases while 2.7% (4/147) were NTM. Of the four patients with NTM, three were diagnosed as tuberculosis by FNA cytology. FNA cytomorphology had classified 80.3% (151/188) of the clinical presumptive TBLN cases as typical TB of which 21 were negative on culture (Table 3).

**Table 3:** **Comparison of cytomorphological changes with bacteriological methods for the diagnosis of TBLN (n = 188)**

Bacteriological methods		FNA cytology result		
		Positive	Negative	Total
**AFB microscopy**	Positive	42	4	46
	Negative	109	33	142
**MGIT 960**	Positive	129	17	146
	Negative	22	20	42
**Glycerol based LJ media**	Positive	114	11	125
	Negative	37	26	63
**Pyruvate based LJ media**	Positive	111	12	123
	Negative	40	25	65
**Composite gold standard (LJ and or MGIT)**	Positive	130	17	147
	Negative	21	20	41

Overall, FNA cytology had a sensitivity of 88.4% and a specificity of 48.8% (Table 4). Similar results were obtained when compared with MGIT 960 alone (88.4% and 47.6%, respectively). MGIT 960 detected more cases as compared to solid culture (LJ media) with 12.3% additional sensitivity. When compared to FNA cytology, the incremental yield of MGIT 960, LJ and AFB microscopy among presumptive TBLN cases was 9%, 6.3% and 2%, respectively. In total, 7.5% (11/147) of all culture-positive cases were diagnosed as ‘non-specific abscess’ by FNA cytology. AFB smear microscopy detected 10.8% (4/37) tuberculosis cases that were diagnosed negative by FNA cytology.

**Table 4:** **Diagnostic efficiency of FNA cytology as compared to composite gold standard (n = 188)**

Diagnostic efficiency of FNA cytology	Value	95% Confidence interval
**Sensitivity**	88.4%	82.1%- 93.1%
**Specificity**	48.8%	32.9% -64.9%
ROC* area (Sens. + Spec.)/2	0.686	0.604- 0.768
**Likelihood ratio (+)**	1.73	1.27 -2.34
**Likelihood ratio (−)**	0.237	0.137- 0.409
**Odds ratio**	7.28	3.32- 16
**Positive predictive value**	86.1%	79.5% -91.2%
**Negative predictive value**	54.1%	36.9%- 70.5%

*ROC = Receiver operator characteristic curves (plot of false positives against true positives for all cut-off value).

Half of culture-negative cases (51.2% (21/41)) were classified as tuberculosis by FNA cytology. Caseous specimens were more likely to be diagnosed as tuberculosis by FNA cytology than purulent specimens (p- value = 0.003). Culture did not reveal any difference in positivity rate based on specimen type (p -value = 0.15). The positive predictive value and confirming power of the macroscopic appearance of FNA was high for caseous specimens, when compared to FNA cytology. There was no difference in terms of diagnostic accuracy between FNA cytology and bacteriological methods among HIV infected individuals (n = 9). The specificity of FNA cytology varied according to sex. In addition, the diagnostic sensitivity of MGIT 960 was higher among males as compared to females (p -value = 0.042), just as the AFB positivity rate: male patients were more likely to be positive than females (p -value = 0.004).

## Discussion

Diagnosis of TBLN mainly relies on FNA cytology, as it is simple, cost effective and less invasive as compared to excision biopsy. Moreover, it is sensitive and requires minimal laboratory infrastructure [12]. However, since it depends on cytological changes but not on bacterial detection, its specificity is confounded by inflammatory reactions other than those caused by tuberculosis [13]. Additionally, it is not possible to differentiate tuberculous and non-tuberculous mycobacterial causes [14],[15].

Among our study participants, the cervical lymph node region was the most affected site which is similar to another study report from the same area [4]. Bacteriological tests confirmed TBLN in 78% of the presumptive cases while 80.3% were confirmed by FNA cytology. Cytomorphological changes on FNA had a sensitivity of 88.4% with a positive predictive value of 86.1%. Even though FNA is highly valuable in terms of its detection rate, three of the four cases due to non-tuberculous mycobacterial lymphadenitis were classified as TBLN. NTM are ubiquitous in the environment and due to better diagnostic tools, the number that is isolated and identified is increasing. Isolation of NTM could be due to patient colonization or sample contamination but it may as well reflect the real etiologic agent of the disease [16]. The role of NTM in causing lymphadenitis is prominent in children and immune compromised individuals, but also immune competent individuals are susceptible [17],[18].

According to the existing Ethiopian guideline for the diagnosis of TBLN, patients will start anti-TB treatment if FNA cytology is suggestive. In cases of non-tuberculous mycobacterial lymphadenitis, this may lead to inappropriate treatment and to a delay in the real diagnosis. In areas where mycobacterial lymphadenitis is prevalent and the HIV burden is high, the role of NTM should be kept in mind when taking a treatment decision.

Among presumptive cases diagnosed as TBLN by FNA cytology and negative by bacteriological methods, females accounted for a higher proportion (13%) than males (7.5%). On the contrary, male patients were more likely to be AFB microscopy and culture positive on MGIT 960. Epidemiological studies of TBLN based on FNA cytology confirmed that females were more affected as compared to males [19],[20]. This could be due to the predominance of non-specific lymph node reactions among females, as well as differences in bacterial load and the poor specificity of FNA cytology [21]. This calls for more specific methods that confirm the presence of the etiologic agent.

Most cases diagnosed as abscess on FNA cytology were culture positive. The MGIT 960 mycobacterial detection system increased the sensitivity of true FNA cytology positive cases by 12% without redundancy. This method is resource intensive and technically complex. However, if the facility allows it, it is highly recommended to consider FNA cytology negative presumptive TBLN cases for liquid culture. An additional advantage is that it allows for subsequent species identification and eventual drug susceptibility testing. AFB microscopy detected 31.3% of culture-confirmed cases. Interestingly, 31% of cases diagnosed as abscess by cytomorphology were AFB positive. Since it is simple and cost effective, supplementing FNA cytology with Ziehl-Neelsen microscopy can improve the diagnosis in patients presenting with lymphadenopathy.

In areas where disease prevalence is high, diagnostic methods with high specificity are important to decrease the probability of false positive diagnoses [22]. In this study the receiver operating curve area, the specificity and the negative predictive value of FNA cytology were low, thereby indicating its poor discriminatory capacity among true negative cases. The pitfall for the pathologist could be other granulomatous reactions in the absence of MTBc. In high TB burden countries like Ethiopia, applying FNA cytology as a sole diagnostic method implies misdiagnosed patients. They may wrongly receive long-term anti-TB treatment, with all its possible side effects, and the true diagnosis may be delayed, or may never be found. Put in a broader way, it may also affect the quality of service to TB cases as resources are going to wrongly diagnosed patients.

Caseous specimens were more likely to be suggestive for TB by FNA cytology as compared to purulent type specimens, corroborating previous reports that caseation is strongly indicative for TBLN [23],[24]. We did not observe a higher yield by culture but purulent specimens were more likely to be positive on AFB microscopy. This suggests that combining AFB microscopy with FNA cytology may increase the specificity of the diagnosis.

## Conclusions

In conclusion, supplementing FNA cytology with AFB microscopy and mycobacterial culture in an endemic region like Ethiopia helps to increase the specificity of the diagnosis of TBLN. Moreover, culture allows drug resistance testing which was hitherto given less attention in this form of extrapulmonary TB. All this may substantially improve the management of patients that present with lymphadenpathy.

## References

[1] World Health Organization(WHO): *WHO report 2012:**WHO Global Tuberculosis report**2012.* Geneva: World Health Organization; 2012.,

[2] Biadglegne F, Tesfaye W, Anagaw B, Tessema B, Debebe T, Mulu A, Sack U, Rodloff AC: Tuberculosis lymphadenitis in ethiopia. *Jpn J Infect Dis* 2013, 66(4):263–268., 10.7883/yoken.66.263 23883834

[3] Federal Ministry of Health of Ethiopia(FMOH): Guidelines for Clinical and Programmatic Management of TB, Leprosy and TB/HIV in Addis Ababa, Ethiopia. 2013.

[4] Abebe G, Deribew A, Apers L, Abdissa A, Deribie F, Woldemichael K, Shiffa J, Tesfaye M, Jira C, Bezabih M, Aseffa A, Bekele A, Colebunders R: Tuberculosis lymphadenitis in Southwest Ethiopia: a community based cross-sectional study. *BMC Public Health* 2012, 12(1):504., 10.1186/1471-2458-12-504 PMC3418151 22770435

[5] Federal Ministry of Health of Ethiopia(FMOH): First Ethiopian natioanal population based tuberculosis prevalence survey. Addis Ababa, 2011.

[6] Iwnetu R, van den Hombergh J, Woldeamanuel Y, Asfaw M, Gebrekirstos C, Negussie Y, Bekele T, Ashenafi S, Seyoum B, Melaku K, Yamuah L, Tilahun H, Tadesse Z, Aseffa A (2009). Is tuberculous lymphadenitis over-diagnosed in Ethiopia? Comparative performance of diagnostic tests for mycobacterial lymphadenitis in a high-burden country. Scand J Infect Dis.

[7] Kidane D, Olobo JO, Habte A, Negesse Y, Aseffa A, Abate G, Yassin MA, Bereda K, Harboe M (2002). Identification of the causative organism of tuberculous lymphadenitis in ethiopia by PCR. J Clin Microbiol.

[8] Biadglegne F, Birhanu A, Debebe T, Tesfaye B, Tessema B, Rodloff AC, Sack U (2013). Tuberculosis lymphadenitis and its treatment outcome in felege hiwot referral hospital, northwest ethiopia: a retrospective study. Int J Biol Med Res.

[9] Yassin MA, Olobo JO, Kidane D, Negesse Y, Shimeles E, Tadesse A, Demissie A, Britton S, Harboe M, Aseffa A, Abate G (2003). Diagnosis of tuberculous lymphadenitis in Butajira, rural Ethiopia. Scand J Infect Dis.

[10] Verma K, Kapila K (2002). Aspiration cytology for diagnosis of tuberculosis–perspectives in India. Indian J Pediatr.

[11] Bezabih M, Mariam DW (2003). Determination of aetiology of superficial enlarged lymph nodes using fine needle aspiration cytology. East Afr Med J.

[12] Giri S, Kar, Singh E: Fine needle aspiration cytology for the diagnosis of tuberculosis lymphadenitis. *UCRR* 2012, 4(24):124–130.

[13] Nataraj G, Kurup S, Pandit A, Mehta P (2002). Correlation of fine needle aspiration cytology, smear and culture in tuberculous lymphadenitis: a prospective study. J Postgrad Med.

[14] Mohapatra PR, Janmeja AK (2009). Tuberculous lymphadenitis. J Assoc Physicians India.

[15] Arend SM, van Soolingen D, Ottenhoff TH (2009). Diagnosis and treatment of lung infection with nontuberculous mycobacteria. Curr Opin Pulm Med.

[16] Wagner D, Young LS (2004). Nontuberculous mycobacterial infections: a clinical review. Infection.

[17] Scott CA, Atkinson SH, Sodha A, Tate C, Sadiq J, Lakhoo K, Pollard AJ: Management of lymphadenitis due to non-tuberculous mycobacterial infection in children. *Pediatr Surg Int* 2012, 28(5):461–466., 10.1007/s00383-012-3068-8 22438045

[18] Chetchotisakd P, Kiertiburanakul S, Mootsikapun P, Assanasen S, Chaiwarith R, Anunnatsiri S (2007). Disseminated nontuberculous mycobacterial infection in patients who are not infected with HIV in Thailand. Clin Infect Dis.

[19] Muluye D, Biadgo B, Woldegerima E, Ambachew A: Prevalence of tuberculous lymphadenitis in Gondar University Hospital, Northwest Ethiopia. *BMC Public Health* 2013, 13(1):435., 10.1186/1471-2458-13-435 PMC3651280 24499165

[20] Fontanilla J-M, Barnes A, von Reyn CF: Current Diagnosis and Management of Peripheral Tuberculous Lymphadenitis. *Clinical Infectious Diseases* 2011,53(6):555–562., 10.1093/cid/cir454 21865192

[21] Tanwir F AI, Hashmi S, Ahmed S: Tuberculosis and Cervical Lymphadenopathy-A Study of 175 Cases in a Tertiary Care Hospital. *Oral Hyg Health 1:119 doi:104172/2332-07021000119* 2013.,

[22] Brenner H, Gefeller O (1997). Variation of sensitivity, specificity, likelihood ratios and predictive values with disease prevalence. Stat Med.

[23] Finfer M, Perchick A, Burstein DE (1991). Fine needle aspiration biopsy diagnosis of tuberculous lymphadenitis in patients with and without the acquired immune deficiency syndrome. Acta Cytol.

[24] Ahmed HG, Nassar AS, Ginawi I (2011). Screening for tuberculosis and its histological pattern in patients with enlarged lymph node. Patholog Res Int.

